# Improved prediction of microbial optimal growth temperatures with neural networks and protein language models

**DOI:** 10.3389/fgene.2026.1874451

**Published:** 2026-07-20

**Authors:** Mensur Dlakić, William P. Inskeep

**Affiliations:** 1 Department of Microbiology and Cell Biology, Montana State University, Bozeman, MT, United States; 2 Department of Land Resources and Environmental Sciences, Montana State University, Bozeman, MT, United States; 3 Thermal Biology Institute, Montana State University, Bozeman, MT, United States

**Keywords:** genome sequencing, machine learning, neural network, optimal growth temperature, protein language models, sequence-based predictions

## Abstract

**Introduction:**

Temperature is one of the strongest selective forces that determines the composition of microorganisms in the environment. Structural properties of proteins shape the thermal adaptation of an organism at the macro level, and aggregate protein features can be used for many types of predictions. Because most microorganisms remain uncultured, the inference of physiological traits, such as optimal growth temperature, has become essential for microbial ecology and biotechnology.

**Methods:**

A large dataset of optimal growth temperatures for microorganisms was compiled from the literature and used to train several machine learning predictors. Our goal was also to test the usefulness of protein language models and to evaluate predictive performance on incomplete genomes.

**Results:**

We confirmed a strong correlation between protein sequence properties and optimal growth temperatures. The analysis showed that calculating better protein sequence features, specifically through protein language models, leads to more accurate predictions.

**Discussion:**

We used state-of-the-art tools and compared our models with several others developed for optimal growth temperature prediction over the past 2 decades. Our models showed excellent ability to generalize across a range of temperatures. We concluded that larger datasets and increased representation of psychrophiles and thermophiles will be needed to continue improving the predictors.

## 1 Introduction

Microorganisms grow across a wide range of temperatures and are grouped into psychrophiles, mesophiles, and thermophiles based on their optimal growth temperatures (OGTs). Their ability to thrive under changing environmental conditions is rooted in physicochemical properties of their biological molecules (Cambillau and Claverie, 2000; Szilagyi and Zavodszky, 2000; Nakashima et al., 2003). The OGT of a microorganism is one of the key physiological traits that reflects its evolutionary and ecological adaptation to the natural habitat. Our lack of knowledge about microbial phenotypes has been exacerbated by the exponential growth of genomic sequence data in this century. Traditional approaches to determine OGT rely on the characterization of pure isolates in the laboratory, but this strategy is impractical for most microbial taxa that cannot be cultivated. Sequence-based predictions offer a viable alternative, enabling OGT estimation directly from genomic information without prior knowledge of culturing conditions (Sauer and Wang, 2019; Karlsen et al., 2023). Importantly, this includes metagenome-assembled genomes (MAGs), which are reconstructed at varying degrees of completeness from environmental samples.

In early studies, genome-derived features obtained from DNA and protein sequences have predicted OGTs with varying degrees of success (Cambillau and Claverie, 2000; Szilagyi and Zavodszky, 2000; Nakashima et al., 2003; Sauer and Wang, 2019; Zeldovich et al., 2007). This was in part due to the initial shortage of data, but also because proteins and DNA do not encode the same type of information when it comes to OGTs. For example, it was shown that the G + C content of rRNA and tRNA correlates well with OGTs, while global nucleotide frequencies and the genomic G + C content do not (Nakashima et al., 2003). Both dinucleotide DNA frequencies and single amino acid frequencies (AAFs) have a strong ability to predict OGTs (Nakashima et al., 2003; Zeldovich et al., 2007), yet dipeptide frequencies (DiFs) are even better (Ding et al., 2004; Li et al., 2019; Gado et al., 2020). Other correlations were noted between the higher charged residue content and increased hydrophobic packing in thermophiles, while psychrophiles show opposite trends (Sauer and Wang, 2019; Zeldovich et al., 2007; Saelensminde et al., 2009; Ebrahimi et al., 2011). It was shown in recent studies that certain classes of non-coding RNAs in Archaea (Arias-Carrasco et al., 2025) and domain frequencies from bacterial genomes (Liu et al., 2025) correlate well with OGTs.

Previous models for OGT prediction were trained on different sets of features, all of which were representations of DNA, RNA, and protein sequences that were extracted on a genomic scale and aggregated (Nakashima et al., 2003; Sauer and Wang, 2019; Zeldovich et al., 2007; Li et al., 2019; Gado et al., 2020; Jensen et al., 2012; Engqvist, 2018; C et al., 2020). In contrast, protein language models (PLMs) encode protein sequences in a different way that correlates not only with sequence properties, but also with structural and evolutionary relationships between proteins (Rives et al., 2021; Elnaggar et al., 2022; Teufel et al., 2022; Singh et al., 2022). This is due to the information-rich nature of high-dimensional PLM vectors (Elnaggar et al., 2022). Latent features of PLMs likely contain other useful information that makes them a method of choice for predicting various protein properties (Elnaggar et al., 2022; Singh et al., 2022), but may also help predictions at the phenotypic level. PLMs predict thermal properties of individual proteins with greater accuracy than feature-based models (Li et al., 2022; Haselbeck et al., 2023; Lv et al., 2025.), which prompted us to consider PLMs for OGT prediction.

Here, we improve the accuracy of models for OGT prediction by leveraging a large training dataset of carefully selected genomes and MAGs. We first built several linear machine learning (ML) models that served as a baseline in our work and a benchmark for comparison with other studies. Next, we added non-linear ML models, including neural networks (NNs), to predict OGTs with high accuracy across a wide range of temperatures. Finally, we used PLM embeddings in combination with NNs to achieve state-of-the-art results. We offer ideas for future improvements in terms of data collection and method selection. A Python package for OGT prediction based on this work is freely available (https://github.com/mdlakic/NeNe-Top).

## 2 Materials and methods

### 2.1 Software

All ML analyses were done using Python v3.8.20. For FASTA sequence manipulations, we developed custom scripts based on BioPython v1.81 (Cock et al., 2009). Three types of datasets were created: 1) single AAFs (20 features); 2) DiFs (400 features); 3) PLM vector representations (1024 features).

Ridge regression (Ridge), lasso regression (Lasso), and support vector regression (SVR) from the scikit-learn package v1.1.0 (Pedregosa et al., 2011) were used for model training. We used fully connected feed-forward NNs with backpropagation as implemented in Keras v2.8.0 (Chollet et al., 2015). NN parameters were as follows: 4 hidden layers (128, 64, 32, and 16 neurons); weights initialization with “he_normal” layers; a single-neuron final layer with a linear activation function; Adam optimizer; batch size of 32; an initial learning rate of 0.01 that was monotonically decayed in each epoch by a factor of 0.005 until it reached 0.001; a total of 5000 epochs with early stopping after 30 epochs if the validation RMSE did not improve. Figures were made using Matplotlib v3.7.2 (Hunter, 2007).

The ProtT5 PLM (Elnaggar et al., 2022) was used to create a high-dimensional vector representation for all protein sequences. PLM embeddings capture many biological signals that are encoded both in local sequence patterns and in long-range relationships. While the embeddings reflect structural tendencies and evolutionary patterns for each amino acid residue (Lin et al., 2023; Erckert and Rost, 2024), their average values can be used to create a single 1024-value vector for each proteome.

### 2.2 Data compilation and processing

**FIGURE 1:**
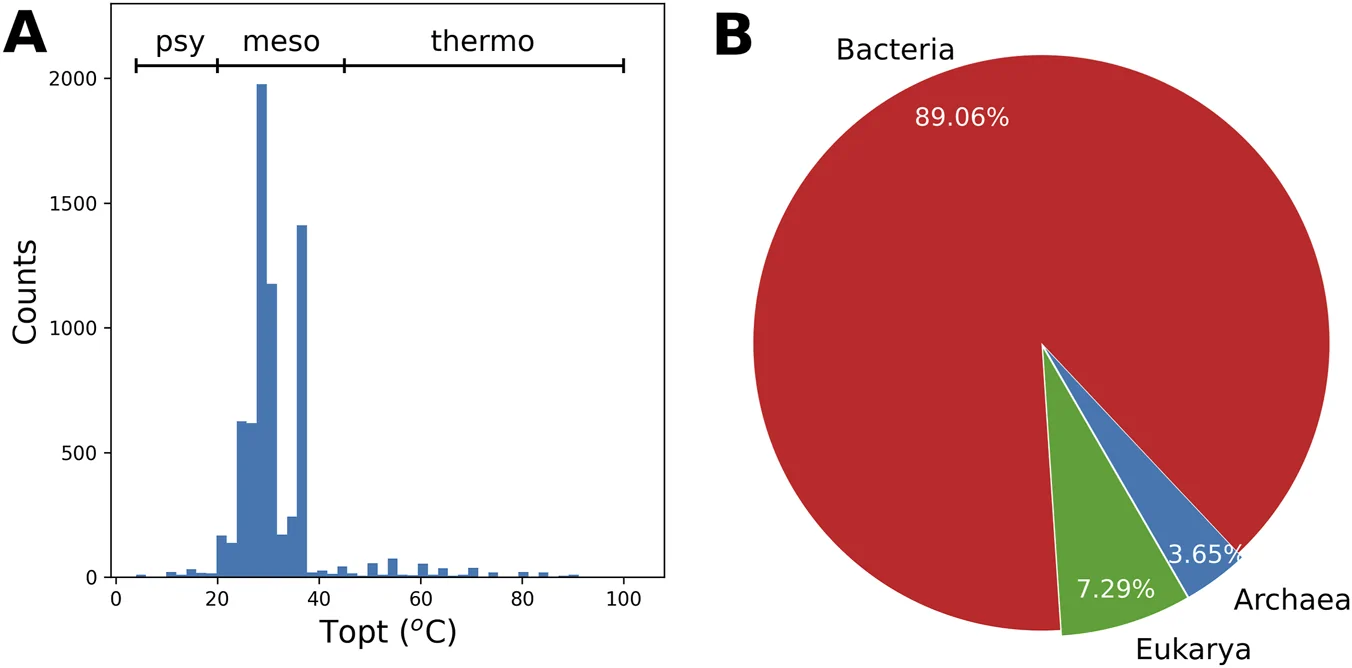
**(A)** A distribution plot of all OGT data points used in this work. As in previous studies, the dataset is skewed heavily towards mesophiles (90.8% of all points), while psychrophiles and thermophiles are represented by 3.3% and 5.9% data points, respectively. Temperature ranges marked at the top are: *psy*, psychrophiles (OGT up to 20 °C); *meso*, mesophiles (OGT 21 °C–45 °C); *thermo*, thermophiles (OGT >45 °C). **(B)** Taxonomic distribution of all OGT data points in three kingdoms of life.

We collected microbial OGT data from the existing literature (Nakashima et al., 2003; Li et al., 2019; Engqvist, 2018; Sato et al., 2020). The initial list had more than 20,000 organisms with OGT values. However, genomic sequences are not available for many of them, and some MAGs were excluded because they were less than 50% complete as determined by CheckM (Parks et al., 2015). We also excluded redundant strains of the same species by retaining the strain with highest CheckM completeness. This was done as their OGTs were likely to be near-identical, and using one of those strains for training and a related strain for testing would bias our results and inflate the final statistics. Our final dataset included 7149 entries that were a mix of complete genomes and MAGs; 6729 were non-thermophiles (OGT ≤45 °C) and 420 were thermophiles (OGT >45 °C). In terms of taxonomic distribution, the dataset contained 261 *Archaea*, 6367 *Bacteria*, and 521 *Eukarya* (Figure 1B).

All the data used for training and testing are available as Supplementary Material. The package we developed for OGT prediction is called NeNe-Top (Neural Network for Temperature Optimum Prediction) and is available from a GitHub repository https://github.com/mdlakic/NeNe-Top under a permissive Apache 2.0 license.

### 2.3 Model training and validation

To get unbiased evaluation of predicted versus observed OGTs, 80% of the dataset was used for training (a total of 5719 entries) and the remaining 20% of the data for testing (1430 entries). The split was stratified to ensure that both datasets had equal representation of entries across the whole temperature range. The test dataset was used only for the comparison of the predicted and measured OGTs. We note that our complete dataset is larger than in previous studies (Nakashima et al., 2003; Sauer and Wang, 2019; Zeldovich et al., 2007; Li et al., 2019), and the test dataset in particular is much larger than before.

Ten-fold cross-validation (CV) was used by partitioning the training dataset into 10 equal parts. Subsequently, 10 different subsets containing 90% of data were used for training, while the remaining 10% of data was used for validation. By averaging results across 10 validation folds, we obtained an unbiased estimate of model’s performance on unseen data. As NNs are known to produce inconsistent results owing to randomness in weight initialization and during optimization (Glorot and Bengio, 2010), for each fold we trained 3 models with different seeds and averaged their scores.

Best SVR parameters were found after 200 sampling runs of Bayesian optimization (Akiba et al., 2019). Best alpha parameters for Lasso and Ridge were found by 10-fold CV. We sampled 200 uniformly spaced alpha values in the (1e-5, 100) range for Lasso, and in the (1e-7, 1) range for Ridge.

Models were evaluated by the coefficient of determination (R^2^) and the root mean square error (RMSE) between the predicted and actual OGT values. R^2^ is the square of the Pearson correlation coefficient and its values range between 0 and 1. RMSE represents the standard deviation of the differences between predicted and actual values, with lower RMSE values being better.

### 2.4 Simulations of incomplete genomes

Incomplete genomes were simulated by randomly removing protein sequences from the test set in increments of 10%, thus creating subsets of our original data that contained 90%–10% of total proteins (Shen et al., 2016). To eliminate the effects of unintentional bias in a given sampling run, this procedure was repeated 10 times for all proteomes in the test dataset.

## 3 Results

### 3.1 Skewed data distribution of OGT values

As reported in previous studies (Li et al., 2019; Gado et al., 2020), the distribution of OGTs in our dataset (Figure 1A) is highly skewed towards mesophilic organisms (labeled “meso” in Figure 1A). This data skew affects the training process, as squared-error regression is a conditional mean function that is weighed by how often samples occur in the dataset. Consequently, the learned regression function will prioritize reducing the error in the more populated areas of the whole distribution, because doing so reduces the total loss more (Gado et al., 2020; Bishop and Nasrabadi, 2006; He and Garcia, 2009). This data imbalance leads to better OGT predictions for mesophiles than either psychrophiles or thermophiles (Sauer and Wang, 2019; Li et al., 2019; Gado et al., 2020). We propose below how to deal with this problem for thermophilic organisms.

### 3.2 Predictions with linear models

Early OGT predictions had to work with smaller datasets and with linear models (Nakashima et al., 2003). It was shown during the early days of genome sequencing that a sum of fractions of seven amino acids (I, V, Y, W, R, E, and L) correlated linearly with OGTs (Zeldovich et al., 2007), and additional genome-derived features further improve predictions (Sauer and Wang, 2019). Even though subsequent studies demonstrated the superiority of more complex, non-linear models (Li et al., 2019; Gado et al., 2020), predictions using linear models on our expanded dataset are included here for fair comparison.

We used regularized linear models, specifically Lasso and Ridge, because they add a penalty on feature coefficients. As this penalty controls model complexity, Lasso and Ridge produce models that generalize well even with high-dimensional datasets. The top part of Table 1 shows the performance of linear models on single AAFs and DiFs. Lasso and Ridge produce similar predictions, and with single AAFs they are on par or better than previous models (rows Ridge-AA and Lasso-AA in Table 1). However, their performance is significantly better with DiFs, with RMSE <4 on both training and test datasets, and with R^2^ at ∼0.85 (rows Ridge-DIpep and Lasso-DIpep in Table 1; also see Supplementary Figures S1 and S2). We attribute this improvement to our larger datasets and to careful regularization via 10-fold CV.

**TABLE 1:** A comparison of linear (top) and non-linear models (bottom) trained on the same datasets. All models work better with dipeptide frequencies (DIpep) than with simple amino acid frequencies (AA). Another general trend is that non-linear models are superior to linear models. Best model in each section is shown in bold letters.

​	Train CV scores		Test scores	
Linear	RMSE	R^2^	RMSE	R^2^
Ridge-AA	5.96	0.64	6.03	0.63
Ridge-DIpep	**3.66**	**0.86**	**3.95**	**0.84**
Lasso-AA	5.96	0.64	6.04	0.63
Lasso-DIpep	3.69	0.86	3.95	0.84

Non-linear	RMSE	R^2^	RMSE	R^2^
SVR-AA	3.81	0.85	3.73	0.86
SVR-Dipep	3.52	0.87	3.50	0.88
NN-AA	3.44	0.88	3.36	0.89
NN-DIpep	3.21	0.89	3.24	0.89
NN-PLM	**2.98**	**0.91**	**2.94**	**0.91**

### 3.3 Predictions with non-linear models

It was shown that non-linear models–namely, decision trees, random forests, and SVR–are better at predicting OGTs, as they capture important non-linear relationships between amino acid frequencies (Li et al., 2019). It is notable, however, that NNs generally have not been used for OGT prediction, despite being one of the premier tools for non-linear modeling. Therefore, in addition to SVR, we chose NNs as a way of broadening the diversity of available predictors.

Both non-linear models (SVR and NNs) make excellent predictions even with single AAFs (rows SVR-AA and NN-AA, Table 1), superior to those obtained using linear models. This performance with single AAFs is better than many previous models using either single AAFs or DiFs, or their combinations with other genomic features (Nakashima et al., 2003; Sauer and Wang, 2019; Zeldovich et al., 2007). Using DiFs further improves both SVR and NN models, with R^2^ values getting close to 0.9 (rows SVR-DIpep and NN-DIpep in Table 1).

**FIGURE 2:**
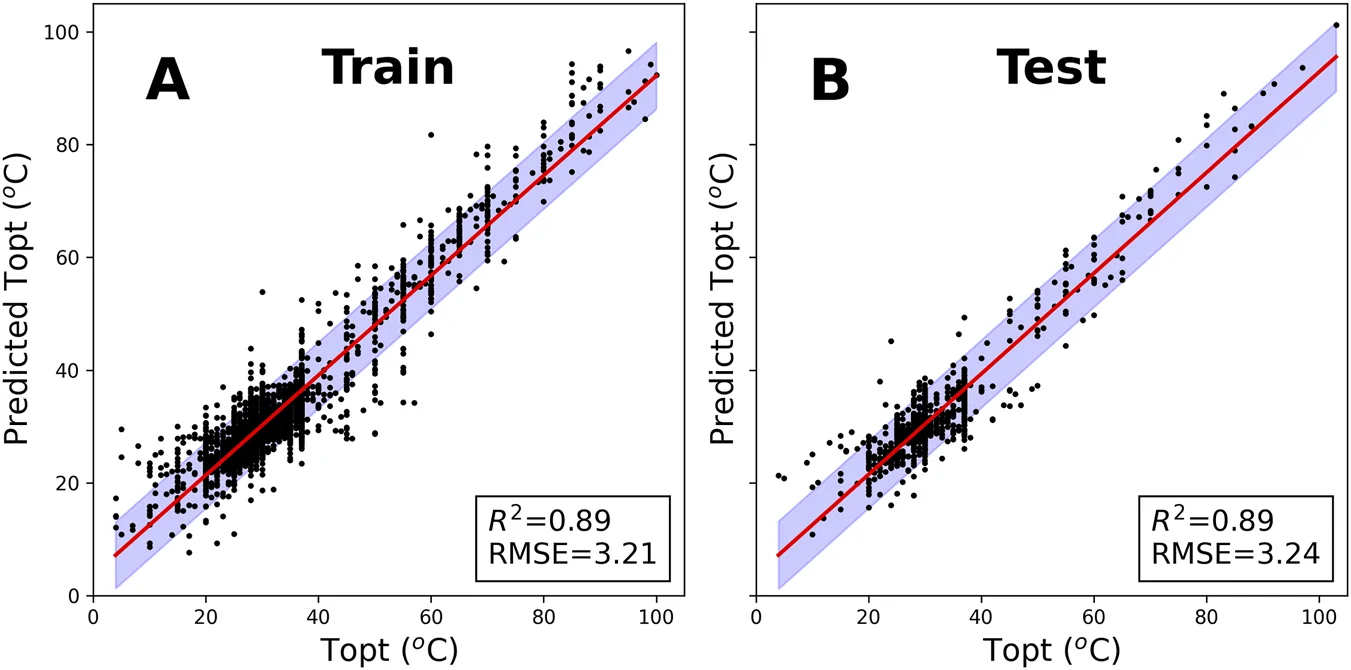
Predictions of a NN model trained on dipeptide frequencies. **(A)** Out-of-fold predictions for the train dataset using 10-fold cross-validation. **(B)** Predictions on the test dataset which was withheld from training. In both figures R^2^ and RMSE are shown in the lower right corner. OGTs are on the X-axis, and predictions are on the Y-axis. Red lines indicate best linear regression fit, while the purple shaded areas around them signify 95% confidence intervals.

The advantage of a large test dataset is apparent when one compares model performance estimates obtained by CV (“Train CV scores” in Table 1) *versus* the actual predictions on unseen data (“Test scores” in Table 1). The agreement between those columns confirms that our models have an excellent ability to generalize. Beyond globally strong predictive metrics, it is important to look at models’ performance across different temperature ranges. A scatter plot between actual training samples and NN predictions (based on 10-fold CV) reveals excellent agreement across a broad temperature range (Figure 2A). The red line represents the best linear fit, while the purple area around it denotes the 95% confidence interval. Most predictions fall within this confidence interval, and there is a similar distribution of points above and below the regression line, which correspond to under- and over-predicted values, respectively. An exception is noted for psychrophiles (OGT <20 °C), whose OGTs are mostly over-predicted (above the red line). A similar pattern, and similar values of predictive metrics, are seen in the test data (Figure 2B). Although they are slightly inferior to NN models, SVR models also predict OGTs uniformly well across the whole temperature range (Supplementary Figure S3).

Since models trained on single AAFs and DiFs perform well, it seemed worthwhile to test a model trained on a combined feature set. Contrary to our expectations, but consistent with a previous report (Li et al., 2019), combining the features did not improve model performance (data not shown).

### 3.4 Predictions with PLMs

PLM representations have been shown to surpass the information content of individual protein sequences and often are competitive with multiple sequence alignments in many prediction tasks (Rives et al., 2021; Elnaggar et al., 2022; Singh et al., 2022; Alley et al., 2019). As high-dimensional PLM vectors encode evolutionary, structural, and functional signals (Elnaggar et al., 2022; Alley et al., 2019), we decided to test whether PLMs can improve OGT predictions. In all previous studies the features were derived from global genomic properties, so we applied the same approach to PLMs. Structural and evolutionary information captured in PLM embeddings was extracted for each amino acid. These numerical matrices were first averaged into a single set of vectors for each protein, followed by aggregation into a single vector for the whole genome.

**FIGURE 3:**
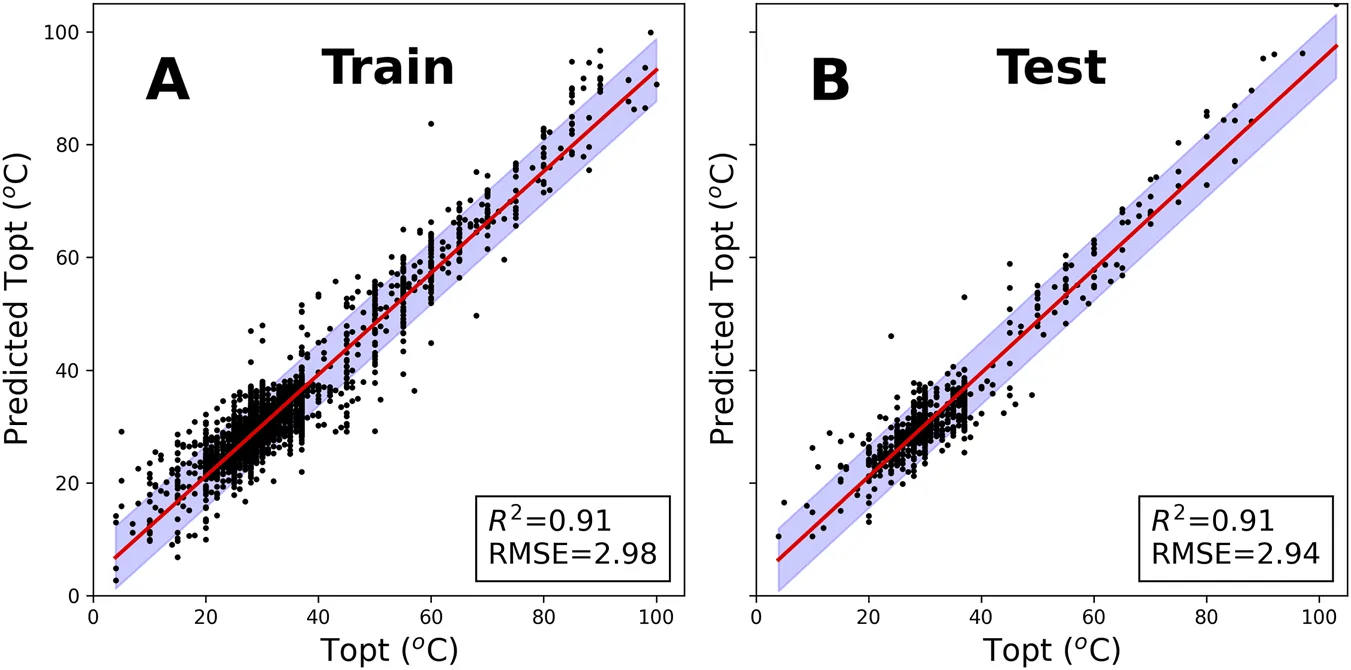
Predictions of a NN model trained on PLM vectors. **(A)** Out-of-fold predictions for the training dataset using 10-fold cross-validation. **(B)** Predictions on the test dataset which was withheld from training. In both figures R^2^ and RMSE are shown in the lower right corner. OGTs are on the X-axis, and predictions are on the Y-axis. Red lines indicate best linear regression fit, while the purple shaded areas around them signify 95% confidence intervals.

The performance of NN models trained with PLM embeddings (last row of Table 1) was superior to other models. For each of the three feature sets, NN models are the best, and perform particularly well when paired with PLM embeddings. To the best of our knowledge, this is the first report where OGTs were predicted with RMSE <3 and R^2^ > 0.9. Importantly, this was achieved both with CV scores and on a large test dataset. As with DiF-based NN models, PLM models make strong predictions across the whole range of temperatures (Figure 3).

### 3.5 Models based only on thermophile data

There is a considerable imbalance in OGT data distribution across temperature as mesophiles dominate among cultivated isolates, and bacteria dominate taxonomically (Figure 1). The latter is less of a problem, as thermal adaptation appears to be uniformly implemented in all kingdoms of life (Li et al., 2022). Unlike early attempts at OGT prediction, our NN models perform with similar accuracy across a wide range of temperatures, as evidenced by high R^2^ scores and narrow distributions of data points around the regression lines in Figures 2, 3. Nevertheless, we were interested in improving OGT predictions for thermophiles, because they are most often subjects of our studies (McKay et al., 2019; Inskeep et al., 2025).

The issue of data skew for OGT was tackled before by combining resampling strategies and ensemble learning (Gado et al., 2020). Resampling often improves R^2^ scores, yet that sometimes comes at the expense of increased RMSE values. We chose instead to train models only with thermophile data (OGT >45 °C). The resulting 420 thermophile data points were divided into 336 training and 84 testing samples. As expected, the NN model performance on thermophilic data had worse predictive metrics, and its CV scores were less correlated with test data (see top portion of Table 2). This can be explained by the small dataset size, as it is more difficult to obtain equal distributions between training and test datasets.

**TABLE 2:** R^2^ and RMSE values for predictions of thermophilic OGTs. Top portion of the table shows predictions by models trained on all available data (general models). Lower portion of the table is based on models that were trained only on thermophilic data (high temperature models). Best model in each section is shown in bold letters.

​	Train CV scores		Test scores	
General models	RMSE	R^2^	RMSE	R^2^
NN-AA	6.47	0.74	5.21	0.84
NN-DIpep	5.93	0.78	4.89	0.86
NN-PLM	**5.24**	**0.83**	**4.12**	**0.90**

High temperature models	RMSE	R^2^	RMSE	R^2^
NN-AA	4.38	0.88	3.99	0.91
NN-DIpep	4.54	0.87	3.98	0.91
NN-PLM	**4.14**	**0.89**	**3.94**	**0.91**

After re-training NN models only with thermophile data, we observed significant improvements for both evaluation metrics (the bottom part of Table 2). Even though the outcome was desirable, we caution users that models based on small datasets may not always generalize well. This is reflected in slightly larger discrepancy between CV metrics obtained from the training dataset and their equivalents from the testing dataset. Our Python package is set to first make a prediction from models trained on the complete dataset. If the first model predicts a value above 45 °C, a second prediction will be calculated automatically based on thermophile data. The two predictions will generally be very similar (see below in Table 3), and it is up the user to choose between them.

**TABLE 3:** Known and predicted OGTs of a select group of thermophilic strains that were isolated in one of our laboratories (WPI).

Organism	Optimal	DIpep	PLM	PLM HT	Reference
Pyrobaculum yellowstonensis WP30	70-82	88.32	90.41	87.42	Macur et al. (2013)
Metallosphaera yellowstonensis MK1	65-75	73.05	72.11	74.84	Kozubal et al. (2012)
Sulfolobales MK5	60-70	69.08	67.07	68.49	Kozubal et al. (2012)
Hydrogenobaculum H55	55-60	61.93	60.20	59.27	Donahoe-Christiansen et al. (2004)

### 3.6 Model performance on a small local subset

As an additional control, we tested the best predictive models on a small group of cultured thermophilic isolates for which the OGT was determined either in the W.P.I. laboratory or by local collaborators (Macur et al., 2013; Kozubal et al., 2012; Donahoe-Christiansen et al., 2004). These isolates were not included in our training dataset to avoid any potential bias. Table 3 shows the experimentally determined OGTs and the predictions by the three best models. There is a small variation between model predictions, but all of them are generally in good agreement with experimental OGTs. A notable exception is *Pyrobaculum yellowstonensis WP30*, which grew best in the 70 °C–82 °C range under laboratory conditions, but all models predict its OGT around 90 °C. This microbe was isolated from outflow channels that had temperatures ranging from 75 °C–85 °C and were near the hot water source spouting at 90 °C (Macur et al., 2013). While this small dataset confirms good predictive ability of our models for thermophiles, we underscore that the results on a much larger testing dataset are more indicative of expected performance.

### 3.7 Model testing on incomplete genomic sequences

During the initial dataset curation, we imposed a criterion of at least 50% genome/MAG completeness, and more than 92% of entries used for both training and testing were >70% complete. Therefore, one would expect our models to work well with highly complete genomes and MAGs, but it was unclear how well they worked on less complete MAGs that are often recovered during metagenomic analyses. We simulated MAGs of varying degrees of completeness by randomly removing 10% of proteins at a time from all test samples, thus creating nine subsets that were 10%–90% complete compared to the original test data. To eliminate bias from random sampling, each incomplete subset of proteins was created 10 times, and their OGTs were predicted independently.

**FIGURE 4:**
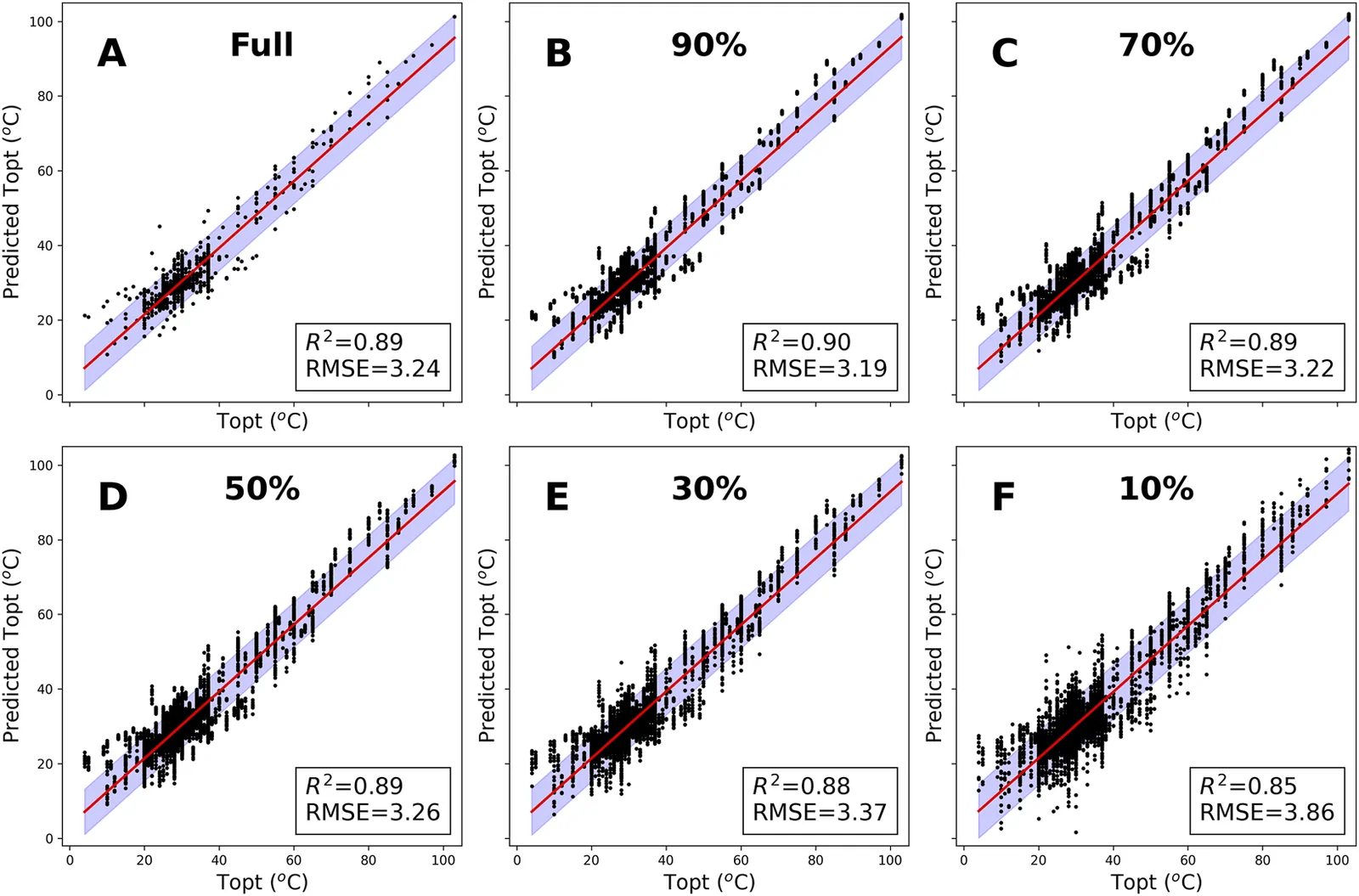
Predictions of a NN model trained on dipeptide frequencies using subsets of test data. **(A)** Predictions on a complete test dataset. This is identical to Figure 2B and is repeated here only for the sake of easier comparison. In panels **(B-F)** the predictions were made on a subset of the test data. Percentage values at the middle top of each panel show how much of the original test data was retained. OGTs are on the X-axis, and predictions are on the Y-axis. Red lines indicate the best linear regression fit, while the purple shaded areas around them signify 95% confidence intervals.

Reducing the number of protein sequences down to 30% had a negligible effect on model predictions, as both R^2^ and RMSE values were essentially the same across the whole range (Figure 4; results for all reduced protein datasets are shown in Supplementary Figure S4). Even at 10%, there is only a small degradation in performance metrics, and the models are still very effective. This finding is qualitatively similar to previous results by Li *et al.* (Li et al., 2019). They noted that OGT predictions show little variation when at least 10^5^ amino acids were included in the training, regardless of the exact protein arrangement in the genome. As the average number of amino acids per genome in our training dataset was 1.65 million, even 10% of that is still more than their minimum cutoff. We conclude that our models make accurate predictions even for MAGs that are 20%–30% complete. Moreover, OGT predictions on MAGs of low completeness could be useful for establishing successful growth conditions that target underrepresented taxa often recovered in environmental metagenomes.

### 3.8 The computational cost

To help in deciding which model to use, we recorded the times needed to make predictions for 1430 entries in the testing dataset. When using DiFs, an average NN prediction takes 5.5 s per MAG. Creating PLM embeddings takes 2.78 min on average, and that step is done separately. A subsequent NN prediction step takes 4.8 s on average. If a prediction >45 °C is made, a second NN prediction is automatically triggered with models trained on high-temperature data, which roughly doubles the total prediction time. All results were obtained by running a single CPU on an AMD Ryzen 9 9950X 16-Core Processor with 192 GB RAM. PLM embeddings were made using an NVIDIA GeForce RTX 4090 card with 24 GB video memory.

## 4 Discussion

### 4.1 The importance of knowing optimal growth conditions

Extremophiles were among the first living organisms, given the hotter conditions on early Earth (Stetter, 2006). Their enzymes were adapted to resist unfolding at high temperatures, but this structural stability made them more rigid and less catalytically efficient at lower temperatures. In contrast, present-day mesophilic enzymes have evolved to be less thermally stable and thus more flexible (Carrea et al., 2000). This evolutionary path led to more optimal catalytic functions, as the pressure to maintain thermal stability was relaxed in moderate environments. The balance of protein stability and catalytic efficiency explains why the fitness of an organism is always relative to its immediate environment, and OGT is a useful proxy for the peak enzyme activity temperature. Therefore, predicting optimal microbial growth conditions from genomic sequences without cultivation is highly valuable for many research and industrial applications.

### 4.2 A comparison of our work with published models

We observed some of the same trends as in previous work: 1) including larger sequence context information (DiFs *versus* single AAFs) leads to better predictions, but combining these two types of data does not result in additional improvements (Zeldovich et al., 2007; Li et al., 2019; Ebrahimi et al., 2011); 2) non-linear models are superior to linear models (Nakashima et al., 2003; Zeldovich et al., 2007; Li et al., 2019; Gado et al., 2020); 3) dataset imbalance hampers predictions, and psychrophiles in particular are poorly predicted (Sauer and Wang, 2019; Li et al., 2019; Gado et al., 2020). However, we also report improvements in each of the areas listed above. Both linear and non-linear models show good performance in our hands, even with single AAFs (Table 1). The availability of larger datasets, especially for rare prokaryotes (Sato et al., 2020), explains a significant reduction in RMSE values compared to earlier work (Zeldovich et al., 2007). Still, linear models trained on single AAFs struggle to make accurate predictions for the whole range of temperatures, which is reflected in relatively low R^2^ scores (Table 1; Supplementary Figures S1 and S2).

Notwithstanding the better overall performance of non-linear models, it is noteworthy that all the models in Table 1 performed well when trained on DiF data. This is a departure from previous studies, where a significant difference was shown in the predictive abilities of linear and non-linear models (Sauer and Wang, 2019; Zeldovich et al., 2007; Engqvist, 2018). Specifically, the performance of Lasso and Ridge with DiFs is very similar to SVR; all models have R^2^ > 0.8 and RMSE <4. There are two likely reasons for this improvement: 1) a large dataset of high quality; 2) thorough parameter optimization for each method. Other groups have trained their models with ∼5000 data points (Li et al., 2019), but in our dataset >92% of genomes/MAGs are at least 70% complete. This strict adherence to high data quality ensures minimal noise during training. Similarly, our test dataset is larger than in previous studies (1430 data points) and shows that our models generalize well. In earlier work, the training might have been done on a relatively large dataset, but the subsequent validation was performed on a much smaller number of entries. Finally, we ran 10-fold cross-validated parameter optimizations for all models to objectively arrive at parameter sets that perform best. Specifically, we tested 200 evenly spaced values for the alpha penalty parameter in Ridge and Lasso. For SVR, we used Bayesian optimization over 200 iterations to find the best combination of its parameters (Akiba et al., 2019). This approach resulted in better models when compared to previous studies.

We emphasize that even though lower RMSE values usually correspond to higher R^2^ scores, these two metrics are not directly interchangeable because R^2^ depends on the total variance in the data. For example, one can observe a high correlation between the actual and predicted OGT values because of the small data spread, yet relatively large RMSE values because of large discrepancies on an absolute error scale (Zeldovich et al., 2007). An inverse result has been reported as well (Li et al., 2019; Gado et al., 2020). Our best NN model trained with PLM embeddings achieves RMSE <3 and R^2^ > 0.9 (Table 1; Figure 3), both of which are high marks in OGT predictions. In addition to rigorous dataset processing and optimization, this accomplishment is attributed to improved data encoding by PLMs. Finally, we showed that our NN models retain essentially the same predictive ability even when small fractions of proteins from the whole genome are used for predictions (Figure 4; Supplementary Figure S4). This high level of accuracy of OGT predictions for relatively incomplete MAGs is one of the most practical contributions of our work, as incomplete MAGs often represent a substantial portion of environmental samples. The success of our models on a large testing dataset inspires confidence that we can now make accurate OGT predictions on genomes of uncultured microorganisms.

### 4.3 Future areas of improvement

Even though we tried to maximize the performance at each step of the process, it is worth contemplating ideas for further improvements. There is a clear trend of better OGT predictions with the increasing number of features: dinucleotides (16 features, of which 10 are unique) < simple AAFs (20 features) < DiFs (400 features) < PLM vectors (1024 features). It is likely that tripeptide frequencies would further improve OGT predictions, because that dataset would better address the neighboring effects by including 8000 unique features. However, the curse of dimensionality postulates that an increase in the feature number requires a disproportionally higher number of training data points (Bishop and Nasrabadi, 2006), which we estimate to be an order of magnitude more than we have right now. This makes the use of tripeptides unlikely in the near future. Some improvement could be expected from PLMs, specifically by finding a subset of proteins from each genome that provides the best PLM signal for training (Liu et al., 2025).

The surest way to improve future OGT predictions, regardless of the method used, is to start with larger datasets that include more psychrophiles and thermophiles. This can be achieved if all scientists who culture microorganisms were to determine and report OGTs. To reduce experimental burden, growth temperatures of cultured isolates are often sampled in 5–10 degree increments. However, as the average error of our best predictor is <3 °C, the measurement error of a 5 degree sampling would unnecessarily decrease signal-to-noise ratios. We therefore encourage scientists to determine OGTs as accurately as possible, preferably by testing growth in 1–2 degree increments.

## Data Availability

The original contributions presented in the study are included in the article/Supplementary Material, further inquiries can be directed to the corresponding author.
