# Supplementary material for: Improved prediction of microbial optimal growth temperatures with neural networks and protein language models
Source: Front Genet. 2026 Jul 20;17:1874451. doi: 10.3389/fgene.2026.1874451 (PMC13454744; doi:10.3389/fgene.2026.1874451)
Supplement: Supplementary file 1 [file Supplementaryfile1.docx]

Supplementary Material for

Dlakić & Inskeep: “Improved prediction of microbial optimal growth temperature with neural networks and protein language models”

# 1 Supplementary Data

The following supplementary files are included:

# 2 Supplementary Figures and Tables

## 2.1 Supplementary Figures

**Supplementary Figure 1.** Predictions of a ridge regression model trained on dipeptide frequencies. **A)** Out-of-fold predictions for the train dataset using 10-fold cross-validation. **B)** Predictions on the test dataset which was withheld from training. In both figures coefficient of determination (R^2^) and root mean squared error (RMSE) are shown in the lower right corner. OGTs are on the X-axis and predictions are on the Y-axis. Red lines indicate the best linear regression fit, while the purple shaded areas around them signify 95% confidence intervals.

**Supplementary Figure 2.** Predictions of a lasso regression model trained on dipeptide frequencies. **A)** Out-of-fold predictions for the train dataset using 10-fold cross-validation. **B)** Predictions on the test dataset which was withheld from training. In both figures R^2^ and RMSE are shown in the lower right corner. OGTs are on the X-axis and predictions are on the Y-axis. Red lines indicate the best linear regression fit, while the purple shaded areas around them signify 95% confidence intervals.

**Supplementary Figure 3.** Predictions of a support vector regression model trained on dipeptide frequencies. **A)** Out-of-fold predictions for the train dataset using 10-fold cross-validation. **B)** Predictions on the test dataset which was withheld from training. In both figures R^2^ and RMSE are shown in the lower right corner. OGTs are on the X-axis and predictions are on the Y-axis. Red lines indicate the best linear regression fit, while the purple shaded areas around them signify 95% confidence intervals.

**Supplementary Figure 4.** Predictions of a neural network model trained on dipeptide frequencies using subsets of test data. **A)** Predictions for the complete test dataset. This is identical to Figure 2B and is repeated here only for the sake of easier comparison. In panels **B-J** the predictions were made on a subset of the test data. Percentage values at the middle top of each panel show how much of the original test data was retained. In all panels R^2^ and RMSE are shown in the lower right corner. OGTs are on the X-axis and predictions are on the Y-axis. Red lines indicate the best linear regression fit, while the purple shaded areas around them signify 95% confidence intervals.
